# Non-Pharmacological Treatment of Primary Headaches—A Focused Review

**DOI:** 10.3390/brainsci13101432

**Published:** 2023-10-08

**Authors:** Emir Licina, Aleksandra Radojicic, Marta Jeremic, Aleksandra Tomic, Milija Mijajlovic

**Affiliations:** 1 Faculty of Medicine, University of Belgrade, 11000 Belgrade, Serbia; licina020@gmail.com (E.L.); aleksandraradojicic@gmail.com (A.R.); alexandra_tomic@yahoo.co.uk (A.T.); 2 Neurology Clinic, University Clinical Center of Serbia, 11000 Belgrade, Serbia; marta.jeremic@gmail.com

**Keywords:** headache, migraine, complementary medicine, nutraceutical, acupuncture, biofeedback, cognitive behavioral therapy, neuromodulation

## Abstract

Headache disorders are a significant global health burden, leading to reduced quality of life. While vast pharmacological treatments are available, they may be associated with adverse effects or inadequate efficacy for some patients, therefore there is a need for exploring alternate treatment strategies. This review gives a brief explanation and evaluation of some established and emerging non-pharmacological approaches for headache management, focusing on nutraceuticals and diet, acupuncture, cognitive behavioral therapy (CBT), biofeedback, relaxation techniques, autogenic training, and neuromodulation. Special consideration is given to psychological interventions as they increase patient self-efficacy and provide strategies for managing chronic pain. Future research should focus on optimizing these therapies, identifying patient-specific factors influencing their effectiveness, and integrating them into holistic headache management strategies.

## 1. Introduction

Headache is defined as any type of pain localized in the region of the head. The international classification of headache disorders divides headache disorders into three main groups: primary headache which is of unidentified etiology, secondary headache with evident cause of the headache and neuropathies, and facial pains and other headaches [1]. Headaches are among the most common neurological complaints today; however, the treatment of these conditions has been a problem of physicians for decades. Current epidemiologic data estimates that prevalence of headache throughout lifetime is 96%, with a female predominance, although gender dominance depends also on the headache type. Tension-type headache is the most common headache type with a prevalence of approximately 40% whilst the prevalence of migraine is about 10% [2]. Headaches still represent a significant health burden worldwide. Migraine, which caused 45.1 million YLDs, was estimated to have a very high disability weight, much higher than tension-type headache which was estimated to 7.2 million YLDs globally in 2016 [3]. Headache disorders are associated with impaired quality of life, substantial loss of productivity, and high economic costs worldwide. Headache has been ranked as the second leading cause of years lived with disability according to The Global Burden of Disease study [4]. 

While the physician in everyday practice has at his disposal a vast array of prescription medications for managing headaches, they may be ineffective or contraindicated in a substantial number of patients. These complex disorders which impact the individual in many areas of daily life require a treatment plan which includes both prophylactic and acute measures. No doubt the existing pharmacotherapy approach is invaluable for treatment, however, non-pharmacologic approaches are gaining popularity in recent years and should be considered as viable options as they can be beneficial adjuncts or alternatives where necessary [5]. That being said, issues related to awareness, knowledge, and treatment beliefs indicate that a substantial number of healthcare providers and patients remain skeptical about specific non-pharmacologic interventions (NPIs) and may lack a complete understanding of their underlying principles or the available evidence-based NPI options for various health conditions, including headache [6]. 

### 1.1. Why Non-Pharmacological Therapy?

There are many reasons for employing non-pharmacological treatment options in headache treatment. Here we name the key points.

1. Avoiding medication overuse headaches. It is well established that drugs used for treatment (non-steroidal anti-inflammatory drugs-NSAIDs, ergotamine, triptans), may themselves cause refractory headaches if used excessively [7]. Since the patient is usually unaware that the cause of the headache is the medication itself, this leads to a vicious cycle of frequent use and search for new and more potent drugs. It is important to note that medication overuse headaches are associated with frequency of use, rather than the potency of the medication [8].
2. Side-effects of certain medications. NSAIDs such as aspirin and ibuprofen may cause gastric ulcers and increase risk of adverse cardiovascular and cerebrovascular events. Triptans, used in migraine therapy, may cause nausea, vertigo, and dry mouth and may lead to cardiovascular complications. Opioid analgesics such as codeine and oxycodone cause constipation, somnolence and nausea, and carry with them risk of abuse and addiction [9]. On the other hand, non-pharmacological interventions, such as physical therapy, acupuncture, or cognitive behavioral therapy, generally have minimal side effects and a lower risk profile.
3. Tolerance towards common pharmaceutical therapy may cause difficulties in chronic headache treatment [10]. Complementary non-pharmacologic methods can lower the need for medications and subsequently reduce chances of tolerance buildup.
4. Contraindications for pharmacological treatment. Certain conditions and patient groups (i.e., pregnant and nursing women, patients with comorbidities such as peripheral arterial disease or hepatic impairment) in which pharmacologic treatment may not be a safe option, can benefit non-pharmacological treatment options [11].
5. Cost-effectiveness. Non-pharmacological interventions often have lower upfront costs compared to prescription medications, making them more economically sustainable. Techniques such as relaxation exercises, stress management, and lifestyle modifications generally do not require ongoing expenses.
6. Personal preference. Some patients may have personal reasons for wanting to avoid drugs and seek natural remedies or complementary medicine interventions.
7. Non-pharmacological treatment combined with pharmacotherapy has been proven to be more effective than using each separately [12]. By combining drug therapy with non-pharmacologic interventions tailored to the individual’s specific needs and headache triggers, it’s possible to target the underlying pathophysiological mechanisms from multiple angles, leading to improved symptom management and quality of life for individuals with headaches [13].

### 1.2. What Is Non-Pharmacological Therapy?

Non-pharmacologic interventions (NPI) can be simply defined as any intervention aimed at improving the health of an individual that does not include medication. This includes methods of complementary and alternative medicine (CAM), which has gained popularity amongst patients with headaches in recent years. The National Center for Complementary and Alternative Medicine defines CAM as “a group of various medical and health care systems, practices and products which are not currently viewed as a part of conventional medicine” [14]. Classically, CAM modalities are represented by traditional medicine methods such as acupuncture, nutritional and herbal medicine, chiropractic and massage therapy, homeopathy and similar treatments. NPI also encompasses established psychological methods such as cognitive behavioral therapy and relaxation and stress management techniques. Neurostimulation, biofeedback and other neuro or physical stimulation methods preformed with devices are also considered non-pharmacological interventions. Often times, educating the patient on the nature of the condition and adequate support from the physician and lifestyle changes may be the simplest and effective forms of help in headache treatment [6].

This list is not exhaustive since there are many non-pharmacologic options, we will consider those with most evidence for efficacy in treatment of headache. 

The aim of this narrative review is to present some non-pharmacological procedures with their proposed mechanisms of action and evidence of efficacy in clinical practice. The focus is mainly on nutraceuticals and diet, acupuncture, cognitive-behavioral approaches, biofeedback and relaxation techniques and neuromodulation procedures since the literature suggests these methods to be the most pervasive and effective.

## 2. Nutraceuticals and Diet

Nutraceuticals are defined as any substance that is a food or part if a food and provides medical or health benefits, including the prevention and treatment of disease [15]. Although evidence of the efficacy of many products remains debatable [16], their use is widespread. Here we will also mention diet modifications that may be of assistance in headache management.

### 2.1. Magnesium

Magnesium has been extensively studied for its potential benefits in acute treatment and prophylaxis of migraine [17]. The ion exerts effects multiple factors believed to be involved in migraine pathophysiology including vascular, neurogenic inflammation and oxidative stress mechanisms. Magnesium has been shown to decrease CGRP and substance P levels thereby lessening the vasodilation and nociceptive transmission [18]. Serotonin released from platelets during a migraine attack is also a potent cerebral vasoconstrictor. Mg^2+^ acting as a calcium antagonist, may block serotonin-induced vasoconstriction and promote vasodilation by blocking calcium sensitive potassium channels on smooth muscle cells [19]. Maybe most importantly, Mg^2+^ ions influence glutamatergic transmission via NMDA receptor blocking and thus help regulate glutamate excitotoxicity and cortical spreading depression [20]. Magnesium, alongside riboflavin and coenzyme Q10 is proposed to improve mitochondrial function and reduce brain energy deficit and oxidative stress in migraine [21]. Multiple studies have found decreased levels of serum magnesium in patients suffering from migraine and tension type headache, therefore supplementation with this ion may prove beneficial [22,23,24]. There are different options for treatment with magnesium, the most common being oral supplementation. Products containing magnesium differ in chemical composition (e.g., magnesium oxide, magnesium citrate) and bioavailability which is important for the therapeutic effects. Magnesium supplementation efficacy is considered level B (probably effective and should be considered for migraine prevention) by the American Headache Society and the American Academy of Neurology [25] and is endorsed by European treatment guidelines for migraine [26], with common recommendations being 400–600 mg of oral magnesium daily. Increased dietary intake of magnesium-rich foods such as oats, whole-grain flour, brown rice, almonds, and pumpkin seeds is also encouraged [27].

### 2.2. Riboflavin

Riboflavin (vitamin B2) is a water soluble vitamin that plays an important role in many biochemical processes including synthesis and recycling of niacin and folate, metabolism of fatty acids in brain lipids and nerve myelination and heme protein synthesis [28]. Most importantly, it is a precursor to two coenzymes, flavin adenine dinucleotide (FAD) and flavin mononucleotide (FMN) which are key actors in the electron transport chain and antioxidative function in the cell. While there is no strong evidence of riboflavin deficiency in migraine patients, several studies have shown its potential benefits in migraine prophylaxis, probably via mitigating oxidative stress [29,30,31]. Riboflavin is currently classified as a level B medication for migraine prevention by the American Academy of Neurology [32], the recommended dose being 400 mg oral daily supplementation.

### 2.3. Feverfew

Feverfew (*Tanacetum parthenium*) has been used for centuries in folk medicine as treatment for fevers, headaches, toothaches and other conditions. Feverfew is a perennial herbaceous plant, originating from the mountainous regions of the Balkans but has been widely cultivated in many regions in the world for its medicinal properties. The plant contains different active compounds, among which sesquiterpene lactones-parthenolides are thought to be the most important. Parthenolides act on platelets, inhibiting their aggregation and release of serotonin. The anti-inflammatory function, through the inhibition of prostaglandin and phospholipase A, is also suggested as a mechanism of action [33]. While there is mixed evidence for its effectiveness, some studies have shown a reduction in migraine frequency in patients treated with feverfew extract [34].

### 2.4. Diet

Common dietary products can play a role in the occurrence of headaches, so dietary regimes that limit the use of certain foods are often useful in therapy. Caffeine, even though it is used as an adjuvant to analgesics (such as acetaminophen or ibuprofen), may cause recurrent headaches if taken excessively. Consuming more than 100 mg of caffeine (in medications or drinks) is a known risk factor for chronic headache [35]. Dietary triggers are established phenomena in migraines. The most common culprits are alcohol, tyramine-rich foods such as aged cheese or cured meat, artificial sweeteners, and flavor enhancers like monosodium glutamate (MSG) and nitrites and nitrates used as preservatives in plenty of foods [36]. Studies in children have shown that the “elimination” or oligoantigenic diets with witch certain substances are excluded from consumption may be effective in migraine prophylaxis [37]. There is evidence that obesity may be associated with migraines, potentially through mechanisms involving adipose tissue inflammatory mediators [38,39], hence there is significant improvement in some patient groups who undergo weight loss programs [40]. Further dietary interventions may include a low-carbohydrate, high-fat ketogenic diet (KD) to help in migraine treatment. The classic KD consists of 4 g of fat for 1 g of carbohydrates and proteins combined, replacing the main caloric source of carbohydrates for fats [41]. Most of the 10 selected studies in the recent systematic review [41] reported that KD reduced the number and severity of migraine attacks in adolescents and adults, with few reported adverse effects. The evidence on the effectiveness of the KD is low, so whether the final effect is due to the treatment remains still inconclusive and needs further research aimed to identify specific groups that could benefit from KD as a complementary therapeutic route to the drug treatment of migraine [42]. 

## 3. Acupuncture

Among the practices of traditional Chinese medicine, acupuncture is one of the most widespread in the world today. The earliest documentation of acupuncture as a developed system of diagnosis and treatment dates back to around 100. BCE [43]. Originally, the method involves the insertion of thin needles into specific points on the body to remove blockages of the life-force (Qi) which travels along 14 meridians and comes to 361 classical points. According to traditional views, Qi circulates and preforms it’s function with together with blood, so if there is stagnation in the flow of Qi, blood also stagnates which results in pain [44]. Western science has distanced itself from the traditional metaphysical views and proposed neurobiological mechanisms through which acupuncture provides relief for many conditions. It has also been shown that acupuncture can have effects even if it is performed in non-classical points (sham-acupuncture), and if stimulation is provided without needles (electricity, heat, pressure). There exists a vast amount of literature exploring the effectiveness of acupuncture [45] in a wide range of conditions such as lower back pain, depression, anxiety, headache, insomnia, arthritis, or allergies [46]. Another related technique is dry needling, which differs from acupuncture in that needles are introduced in painful areas and myofascial trigger points as opposed to standardized points. The method has been found to be effective in treating tension and cervicogenic headaches in several studies [47].

Modern theories of the mechanism of action of acupuncture in headaches are based on inducing endogenous analgesia, inhibition of pain processing and mitigating neuroinflammation through peripheral and central pathways. Most of the acupuncture points on the head are found along terminal or cutaneous branches of the trigeminal nerve and between muscle branches of the facial nerve, therefore, their stimulation modulates transmission through pathways important for nociception and neurogenic inflammation [48]. According to the gate control theory of pain, insertion of the needles stimulates delta fibers which close the nerve “gates”, so that nociceptive signals are not transmitted to the thalamus (non-painful input overrides painful input). Especially important is the inhibitory effect on the caudal trigeminal nucleus, which receives pain afferents from cranial blood vessels and the dura [49]. Inflammatory neuropeptides, notably CGRP and substance P, activate the trigeminovascular system, leading to extravasation, dilatation of cranial blood vessels, and increased sensitive conductivity. Biochemical evidence implies that acupuncture modulates the activity of the serotonergic, endocannabinoid, opioid system and endorphin release, and lowers concentration of the mentioned inflammatory mediators [50].

Besides the proposed neurochemical mechanisms, the potential benefits of acupuncture may also be attributed to psychological factors such as positive expectations and prior beliefs about the procedure by the patient, counselling and intensity of care provided by the practitioner and possible placebo effects [51]. In migraine, a recently updated Cochrane review analyzed 22 randomized trials including 4985 participants in total and concluded that adding acupuncture to symptomatic treatment of attacks reduces the frequency of headaches [52]. An updated Cochrane review on acupuncture in tension headache also showed significant improvement in the short term, However, it stressed the need for more quality research [53].

With exact mechanisms of action yet to be fully elucidated, clinical studies have shown acupuncture to be effective in treating and preventing tension-type headache through decreasing pain intensity, duration, emotional comorbidity and medication intake frequency [54]. The procedure is also among the non-pharmacological methods which is recommended by the National Institute of Clinical Excellence in the treatment of tension headache [55].

## 4. The Biopsychosocial Model and Cognitive Behavioral Therapy

Chronic pain, which is present in people suffering from primary headaches is a debilitating condition which impacts the individual in many aspects of life. Migraine sufferers are often unable to get on with daily activities during an attack, which may last up to three days, and seek reclusion in a quiet and dark space. Additionally, in the days preceding the attack, individuals may experience fatigue, changes in mood including increased irritability and stress, and disruption in sleep quality [56]. Patients with episodic and chronic migraine have been shown to be prone to anxiety and feelings of helplessness [57]. On the other hand, patients with cluster headaches exhibit completely different behavior in response to the attack. In most cases these individuals become restless and agitated, they move constantly to try to relieve pain. Sometimes they engage in self-harm, which, most drastically, may lead to attempts of suicide (“suicide headache”) [58]. Tension-type headaches are exacerbated by psychological stress and depression, or may be caused by maintaining inadequate posture for prolonged periods of time (i.e., working with computers) [59]. From these observations follows the question of how behavior impacts headache and vice versa.

The classical understanding of chronic pain solely as a neurophysiological phenomenon is limited and fails to explain why pharmacologic therapy does not provide consistent relief of symptoms or why different individuals react differently to the same medication. Furthermore, complex states such as chronic migraine require considering the whole individual and his lifestyle embedded in his sociocultural setting, since the condition can affect the person in many aspects of social functioning. This is why there is a need for a wider theoretical underpinning in treatment which will complement pharmacological or non-pharmacological interventions. 

The biopsychosocial model offers such a view of chronic pain. This model recognizes that pain, along with any chronic illness, arises from a multifaceted interplay of biological disposition, psychological traits, and social and cultural environments which shape the perception and understanding of pain states. According to the paradigm, the psychological aspects are composed of behavioral, affective and cognitive influences [60]. The behavioral component considers different types of learning which shape the experience and reaction to pain. An example is that of respondent conditioning where acute pain (unconditioned stimulus) is associated with sympathetic activation and increased muscle tension (unconditioned response). With repeated pairings, previously neutral stimuli (environment, body position), which become conditioned stimuli start to elicit fear of pain and sympathetic activation [61]. Affect can be a correlate of pain, a triggering or an exacerbating factor in the occurrence and endurance of pain [62]. Following the Neuromatrix Theory of Pain [63] we see that cognitive functions such as attention, memory, expectations and beliefs, and coping mechanisms play an important role in the experience of pain. Reducing focus on noxious stimuli has been observed to elevate the pain threshold. Conversely, directing attention towards painful stimuli has been found to amplify the perceived intensity of pain, modulating activation of the periaqueductal grey. In fact, distraction techniques are commonly employed as supplementary measures in pain management [64]. 

Coping with pain and patient beliefs about the outcome of their condition and treatment also has impacts on their general functioning. Headache patients tend to use maladaptive thought patterns such as withdrawal or avoidance. Expectation alone may produce headache or influence pain severity [65].

Cognitive behavioral therapy (CBT) is a psychotherapeutic method which relies on the biopsychosocial model and is comprised of various cognitive and behavioral strategies which can be used to improve headache management and quality of life [66]. The cognitive aspect focuses on recognizing dysfunctional beliefs and thought patterns which can generate stress and thus trigger headaches (i.e., catastrophizing, avoidance, and rumination) and replacing them with beneficial ones (acceptance, mindfulness). Behavioral strategies are employed to expose and modify habits and behavior which precipitate, worsen and prolong headaches (smoking, physical inactivity, unhealthy sleeping patterns, diet, etc.) [67]. There are various forms of CBT with treatment modalities ranging from daily sessions preformed at clinics to minimal contact protocols, according to patient needs [68]. The emerging Acceptance and Commitment Therapy (ACT) which emphasizes acceptance and valued living and aims at promoting psychological flexibility is recognized as a potentially beneficial procedure which overcomes some problems associated with classical behavioral interventions aimed at avoiding triggers (lifestyle restriction and decreased locus of control) [67,68]. CBT interventions have been studied in migraine and other primary headaches extensively, and while there are well established and empirically supported behavioral treatments, these approaches remain inaccessible to most patients [69,70,71,72]. 

Patient education can also be of use in managing primary headaches, especially migraine. Therapeutic patient education can be defined as a form of instruction delivered by specially trained healthcare professionals to patients with the aim to equip them with knowledge about their condition to increase self-efficacy and preserve quality of life [72,73]. A systematic review of therapeutic education intervention found evidence of intermediate to long-term disability improvement, quality of life, and migraine frequency [74].

## 5. Biofeedback Training

Biofeedback training is a self-regulation technique with which an individual gains voluntary control of otherwise autonomic functions (muscle tension, heart rate, respiratory rate, skin temperature, etc.) utilizing electroencephalography, electromyoneurography, electrocardiogram or cutaneous thermometers. This intervention requires specialized equipment and a trained professional to guide the patient through training. The procedure is based on registering values of some physiological process, converting and sending the information back (feedback) to the user through audio or video signals. The user, with instructions of a specialist, over multiple sessions gains adequate skills of self-regulation and gradually reduces reliance on the device [75].

The method functions through the principles of operant conditioning through positive reinforcement, where the desired behavior is the change of some physiological variable, and reinforcement is achieved through audio or video display signals and the users conscious involvement [76]. For example, sensors are connected to the skin and register temperature, the information is then converted to indicate the value as red (for warmer) and blue (for colder) on a display. If the patient is relaxed, vasodilation in the skin and the subsequent rise in temperature will show as a red signal on the display, while lowering of the temperature due to reduced blood flow (if the patient is under stress) will cause a blue signal. The patient follows the feedback information and attempts to relax, that is to achieve the desired feedback signal. The same effect can be achieved with audio signals where lower frequencies indicate warmer and higher frequencies indicate colder skin temperature.

In the context of headache treatment, thermal biofeedback is used for migraine and EMG biofeedback is used for tension headaches.

### 5.1. Thermal Biofeedback

Thermal biofeedback, specifically hand warming, operates on the principle that stress, such as a migraine episode, causes peripheral vasoconstriction resulting in cold fingers (presumed vasoconstriction in the peripheral and autonomic nervous systems) [77]. Observations have shown that patients who successfully use biofeedback to induce relaxation and focus on warming their hands experience fewer migraine attacks and reduced headache intensity [78]. A sensor typically attaches to the first finger, measuring the temperature displayed as a numeric value or a line graph depicting temperature fluctuations. Patients monitor their physiological response and employ relaxation techniques to reach their desired temperature goal. Deep breathing, alternating muscle contraction/relaxation, music or autogenic hypnosis using positive phrases are possible ways to achieve relaxation [79].

### 5.2. EMG Biofeedback

EMG biofeedback is based on the use of an electromyogram to detect the tension of the head muscles and the application of relaxation and stress management techniques to eliminate that tension. Classically, electrodes are placed in the area of the frontalis, masseter, and trapezius, as these muscles are often inadequately activated due to improper jaw clenching habits or a hunched body position and stress [73]. Muscle tension is represented by low and high frequency tones for lower and higher tension, respectively. The purpose of EMG biofeedback is for the user to learn over time to recognize triggers that lead to tension, change his reaction and apply relaxation techniques, which results in reduced frequency and intensity of headaches [75].

A 2008 comprehensive review found medium-to-large mean effect sizes for biofeedback in migraine and tension type headache with improvements in headache frequency, medication consumption and in measures of self-efficacy, anxiety and depression symptoms [80]. A 2010 review suggests that diverse forms of biofeedback prove effective in managing both migraine and tension-type headaches. Furthermore, the results achieved through biofeedback are comparable to those attained through medication therapy and combining biofeedback with medication can further improve outcomes. However, despite its efficacy for many patients, a significant portion of headache patients still do not experience substantial relief from biofeedback [81].

## 6. Relaxation Techniques

Relaxation techniques can be practiced and learned in order to regulate the reaction to stress and pain, reduce sympathetic arousal and muscle tension. The methods used to achieve a state of relaxation are very diverse, from traditional yoga and meditation practices to modern variants of breathing exercises and mindfulness. Some proven methods include progressive muscle relaxation, autogenic training and meditation.

### 6.1. Progressive Muscle Relaxation

Progressive muscle relaxation (PMR) was originally introduced by Edmund Jacobson in 1938 to demonstrate the correlation between muscle tension and mental activity, and how systematic relaxation can reduce mental activity [82]. The technique involves achieving a state of “extreme relaxation” by sequentially tensing and then relaxing different muscle groups, with the patient focusing on the contrasting sensations of tension and relaxation. Once the initial phase is learned, additional skills such as recall relaxation, signal-controlled relaxation, and differential relaxation (maintaining relaxation in muscles not actively engaged) are taught. Patients can typically master progressive relaxation training in fewer than 10 sessions. While initially learned in a calm environment, these techniques can be subsequently applied in everyday life. This method has been employed in the treatment of phobias, anxiety, and related disorders, and there are now internet applications available that offer exercise instructions for the treatment of migraine [83]. Several studies indicate that progressive relaxation reduces the frequency of migraines and diminishes the duration and intensity of both tension headaches and combined headaches [84].

### 6.2. Autogenic Training

Autogenic training (AT) is a relaxation technique and a psychophysiological mode of psychotherapy based on passive concentration on body perceptions (i.e., weight or warmth of legs and arms, heartbeat or breathing) facilitated by autosuggestion [85]. Originally, the training is performed sitting or lying down, where the person concentrates on a certain part of the body or a vital function and repeats phrases such as “my right arm is heavy”, “my forehead is cold” (there are six standard exercises according to Schultz) with the aim of achieving deep relaxation [85]. The advantage of this method is that it is relatively simple and can be performed at home every day. Autogenic training has been studied in various chronic pain conditions and there is sufficient evidence to support its effectiveness in improving quality of life for many patients [86,87]. Regular practice of AT for headache has been shown to reduce the frequency of migraines with an observed effect on brain areas associated with aversive emotional processing, emotional stress processing and pain perception [88].

One mechanism that explains the action of the aforementioned relaxation methods is the initiation of the so-called “relaxation response” which has the opposite effect to the stress reaction (“fight or flight” response) [89]. The relaxation response consists of a reduced rate of respiration and heart rate, a decrease in frequency and an increase in the amplitude of brain waves, an increase in body temperature, a decrease in muscle tension and a consequent decrease in the level of anxiety and stress [90]. Regular implementation of these techniques can provide benefits to individuals with headaches by enabling better stress regulation and, consequently, addressing the factors that contribute to headache occurrence [91].

## 7. Physical Therapy

Physical therapy commonly employs cervical manipulation and mobilization, soft tissue therapy such as compression or strokes, therapeutic exercise, and needling techniques in the treatment of different types of headache [92]. 

Joint manipulation denotes quick, controlled movements at the end of joint motion, while mobilization uses slower, more extensive movements within the joint’s normal range. Spinal and cervical manipulation or mobilization is used based on the observation that individuals with headaches experience neck pain and increased tenderness around the head is present in adults with migraine and tension-type headache (TTH). New data suggests that physical therapy in tension-type headache leads to improvement of headache intensity and frequency not only short term (initial time until endpoint was up to 8 weeks) but also long term, for a period of up to 26 weeks post-intervention [93]. However, adverse responses to spinal manipulative therapy are a concern, encompassing mild effects like stiffness, pain and restricted range of motion to more severe instances of permanent neurological impairments or the occurrence of carotid or vertebral artery dissections [92].

Exercise-induced hypoalgesia, linked to the activation of pain-inhibiting pathways, aligns with the idea that managing headaches should go beyond addressing local tissue issues and involve strategies to normalize central nervous system excitability. However, because various mechanisms underlie headaches, the most suitable exercise program may vary for each type. For example, aerobic exercise is recommended for migraine prevention, while specific neck and shoulder exercises are more suitable for tension-type headaches and cervicogenic headaches. Inclusion of cranio-cervical flexion exercises has been shown to effectively reduce headache frequency, intensity, and duration, supporting this hypothesis [92,94]. Although physical exercise has been reported as a triggering factor for migraine, regular exercise may have prophylactic effect on migraine frequency. This is most likely due to an altered migraine triggering threshold in persons who exercise regularly. Additional mechanisms for migraine prevention by exercise may include increased beta-endorphin, endocannabinoid and brain-derived neurotrophic factor levers in plasma after exercise. Therefore migraineurs should be encouraged to practice physical exercise with controlled intensity, frequency and duration to achieve the most beneficial outcome [95].

## 8. Neurostimulation

Neurostimulation, or neuromodulation, is a new and developing field of treatment for chronic pain disorders, including headache. These methods involve the use of electrical or magnetic stimulation to modulate the activity of the brain, cranial and peripheral nerves, with the aim of influencing the pathways for the transmission and perception of pain [96]. Noninvasive neuromodulation techniques have gained popularity in recent years due to their relative safety and ease of use, making them an attractive option for patients who may not be candidates for more invasive procedures. In this regard, noninvasive neuromodulation techniques are increasingly used in the treatment of primary headache, including migraine, tension-type headache, and cluster headache. This approach to treatment is a promising area of research, and ongoing studies are providing insight into the mechanisms of action and efficacy of various neuromodulation techniques.

### 8.1. Transcranial Magnetic Stimulation (TMS)

Transcranial magnetic stimulation (TMS) is a method used in the diagnosis and therapy of various diseases, including multiple sclerosis, Parkinson’s disease, dystonia, and depression. TMS can be used to measure nerve conduction, facilitate or inhibit the electrical activity of the cortex via a magnetic field created by an electric current through a coiled tube wrapped around the head [97]. Magnetic fields are generated in short pulses, hence there is single-pulse TMS, paired-pulse TMS, and repetitive TMS. Single-pulse TMS (sTMS) works by disrupting cortical spreading depression- a wave of cortical hyperexcitability followed by depressed neural activity which is believed to be responsible for migraine aura, pain and sensory changes through activating the trigeminovascular and trigeminocervical systems. Short pulses of TMS (1s) timed at the start of the headache can stop the pathological excitation and thus end the headache or make it less severe [98]. There is evidence that early treatment of migraine with aura with sTMS results in increased and sustained freedom from pain in acute attacks [99]. Repetitive TMS (rTMS) supposedly works in migraine prophylaxis through effects on neurotransmitters such as dopamine or glutamate [100] and has also been reported to increase beta-endorphin levels which is corelated with migraine relief [101]. A 2004 pilot study investigated high-frequency rTMS applied over the left dorsolateral prefrontal cortex, based on observations of the inhibitory effects of this area in pain pathways. The results showed significant improvement in measures of attack frequency and number of abortive medications after 12 sessions in patients with chronic migraine [102]. One hand-held portable TMS device is currently approved by the US FDA for the treatment of migraine with aura [103].

### 8.2. Transcranial Direct Current Stimulation (tDCS)

tDCS is a method of non-invasive brain stimulation based on delivering a weak current through electrodes fixated on the scalp which are connected to a battery stimulator. The procedure achieves modulation of resting membrane potential of neural fibers via anodal stimulation inducing depolarization or cathodal stimulation inducing hyperpolarization [104]. A proposed mechanism of action in is that the current is able to regulate altered functional connectivity of the cortex with subcortical structures (especially the thalamus) in migraine patients [105]. The procedure has been applied with electrodes targeting different brain areas such as the primary motor cortex, the primary sensory cortex, the primary visual cortex and the dorsolateral prefrontal cortex in a number pilot studies with varying technical parameters and number of sessions, therefore there is not yet a standardized protocol [106]. While tDCS has been found to be effective in preventing migraine in a number of RCT’s and shows great potential due to its ease of use there is a need for further research and a consensus for optimal protocol [107]. 

### 8.3. Non-Invasive Vagus Nerve Stimulation (nVNS)

Non-invasive vagus stimulation (nVNS) can be performed using hand-held, portable devices such as the gammaCore^®^ that the patient uses himself by placing the device on the neck in sessions of about 15 min (daily or as needed) [108]. The mechanism of action of nVNS is not completely clear. It is believed that, through the stimulation of the vagus branch, it affects the nucleus tractus solitarii and from there (activating or inhibiting) descending structures such as the thalamus, hypothalamus, reticular activating system and other cores and reduces the transmission of painful stimuli [109]. nVNS is used in the therapy of cluster headaches and migraines according to National Institute of Clinical Excellence recommendations [108]. A recently published systematic review and meta-analysis of randomized controlled studies in patients with migraine found that nVNS can significantly reduce migraine or headache days. Cervical nVNS for migraine markedly increased ≥50% responder rate, and low-frequency auricular nVNS could significantly reduce headache intensity. The findings support that the potential of nVNS to reduce disease burden and improve quality of life in migraineurs [110].

### 8.4. Transcutaneous Supraorbital Nerve Stimulation (tSNS)

Transcutaneous suborbital nerve stimulation (tSNS) is a method of modulating the supraorbital and supratrochlear nerve via an adhesive electrode placed on the forehead (supraorbital). The FDA-approved Cefaly^®^ device is in use. The device is intended for a 20 min session, once a day, every day as a preventive treatment. As the supraorbital and supratrochlear nerves are terminal branches of the frontalis nerve and this one is a branch of the trigeminus, it is considered that the therapeutic effect is mediated by inhibition of nociceptive transmission and modulation of nociceptive activity in the trigeminal ganglion. This method has proven to be effective in migraine prevention [111].

### 8.5. Sphenopalatine Ganglion (SPG) Stimulation

The importance of the sphenopalatine ganglion has become evident after detailed research of cluster headache and other autonomic trigeminal cephalalgias (TAC). The SPG plays a role in headache pain and cranial autonomic symptoms in TACs. The activation of the trigeminal-autonomic reflex is believed to be the main cause of pain generation. Postganglionic parasympathetic fibers that innervate cerebral and meningeal blood vessels are activated and release neuropeptides that trigger trigeminal nociceptor fibers and are perceived as referred pain. Neurostimulation of the SPG causing inactivation of SPG can be managed by an implanted SPG stimulator placed with a minimally invasive and well tolerated surgery which has approval for treatment of cluster headache in the European Union. Results of trials have shown that two-thirds of patients were responders to SPG neurostimulation [112]. 

### 8.6. Deep Brain Stimulation (DBS)

Deep brain stimulation in the sphere of chronic headache syndromes has found its valuable place in the treatment of TACs with hypothalamic stimulation. Over the last decade, a number of clinical trials were conducted in order to evaluate the efficacy of DBS in the treatment of TACs and the results showed a response rate of 50–70%. Even though the efficacy was satisfactory the relapse rate was also remarkable and since DBS is an invasive procedure intraoperative and postoperative complications were observed. Therefore, it is recommended that hypothalamic DBS should be proposed in chronic intractable TACs only after other, less-invasive, neurostimulation procedures have been unsuccessful [113].

Table 1 summarizes non-pharmacological treatment options, relevant references to the type of non-pharmacological treatment, their effectiveness and level of recommendation.

## 9. Conclusions

Since headache represents a complex condition affecting biological and psycho-social well-being, treatment should be based on a multi-faceted approach. There is a growing body of evidence for the efficacy of non-pharmacological interventions either as alternatives or adjuncts to standard drug treatment. Complementary and alternative medicine methods, such as acupuncture, nutraceuticals or herbal medicine, provide solutions which may be attractive to patients due to their relative inexpensiveness and application outside of a hospital setting. That being said, these methods are not yet a part of mainstream medicine and lack sufficiently congruent evidence for broader clinical implementation. Psychological and behavioral strategies including psychotherapy, biofeedback and relaxation techniques help to increase patients self-efficacy, reduce stress and improve mental health status which is important in management of a chronic condition such as headache pain. These strategies require specialized personnel which may limit their availability. Physical therapy may be a valuable addition to headache management mostly in prophylaxis, However, there is still no consensus on standardized protocol. Neuromodulation procedures are advantageous since they target very specific mechanisms underlying headache, especially migraine, and the prospect of portable devices used by patients shows great promise. However, the costs related to the technological equipment and availability limit their broader implementation.

More patients and healthcare providers should be made aware of the alternatives to medication in order to offer a wider range of treatment possibilities, especially to combat excessive use of medications with its consequences. Further research is needed to elucidate fully the mechanisms by which these interventions function in order to deduce indications for application, optimal treatment plans and realistic expectation of outcomes for individual cases. We believe that a diverse approach is a valuable asset in headache management and non-pharmacological options should be considered more in subsequent studies in this field. This article provided an explanation of mechanisms of action of non-pharmacological interventions and evidence of their efficacy, However, it is not a systematic review and hence many other treatment options and studies were not considered.

## Figures and Tables

**Table 1:** Summary of non-pharmacological treatment options for primary headaches.

Type of Non-Pharmacological Treatment	Relevant Referance Numbers	Level of Recommendation
Magnesium	[17,18,19,20,21,22,23,24]	probably effective (level B)
Riboflavin	[27,28,29,30,31,32]	Probably effective (level B)
Feverfew	[34]	Possibly effective
Dietary regimens and ketogenic diet	[35,36,37,38,39,40]	Possibly effective
Acupuncture	[43,44,45,46,47,48,49,50,51,52,53,54,55]	Possibly effective
Biopsychosocial model	[60,61,62,63,64,65]	Possibly effective
Cognitive behavioral therapy	[66,67,68,69,70]	Possibly effective
Biofeedback training	[71,72,73,74,75,76,77,78,79,80,81]	Possibly effective
Relaxation techniques	[82,83,84,85,86,87,88]	Possibly effective
Physical therapy	[92,93,94,95]	Possibly effective
Neurostimulation (TMS, tDCS, nVNS, tSNS, SPG Stimulation)	[97,98,99,100,101,102,103,104,105,106,107,108,109,110,111,112,113]	Probably effective (FDA approved)

TMS—Transcranial Magnetic Stimulation; tDCS—Transcranial Direct Current Stimulation; nVNS—Non-Invasive Vagus Nerve Stimulation; tSNS—Transcutaneous Supraorbital Nerve Stimulation; SPG—Sphenopalatine Ganglion; FDA—Food and Drug Administration.

## Data Availability

The data that support the findings of this study are available on request from the corresponding author.
